# Prevalence and determinants of internet addiction among children with depression: A study in a school setting in Tunisia

**DOI:** 10.1192/j.eurpsy.2024.830

**Published:** 2024-08-27

**Authors:** K. Mayssa, H. Imen, K. Ali, B. Jaweher, B. T. Donia, A. Hela, K. Khaoula, M. Yousr

**Affiliations:** ^1^Department of Child Psychiatry, University Hospital of Hedi Chaker, Sfax, Tunisia

## Abstract

**Introduction:**

Child depression is a public health problem. Its association with internet addiction (IA) could increase the frequency of complications and have a significant impact on the child’s psychological well-being, schooling, family and social life.

**Objectives:**

To study the clinical profile and associated factors of IA in a population of primary school children with depressive disorders.

**Methods:**

This was a descriptive and analytical cross-sectional study of 182 children with depression attending four primary schools in the government of Sfax. The study took place from 1 March 2022 to 30 June 2022. In the present study, we administered the following psychometric scales: Internet Addiction Test (IAT), Revised Children’s Anxiety and Depression Scale (RCADS- 47), Birleson Depression Scale Questionnaire and the Rosenberg Self-Esteem Scale.

**Results:**

The mean age of the children studied was 9.9 ±1.17 years and the sex ratio was 0.8. Mean score of Internet Addiction Test was 40 ±4,46. In addition, Internet addiction was identified in 73.6% of students with depression (N=143). Following a univariate analysis, internet addiction among depressed Childrens was positively correlated to individual factors such as the absence of leisure activity, the number of hours per day spent on the internet (1.63 VS 3.25, P < 0.001) and interest in accessing Tiktok (p=0.002). Internet addiction in depressed children also depended on family factors. Internet addiction was more common among childrens with a medium to high family socio-economic level (P < 0.001) in cases where parent-child communication was deemed unsatisfactory (P = 0.002) and in cases of verbal violence (P < 0.001). We were also able to establish a significant link between internet addiction among depressed pupils and significant symptoms of anxiety (P=0.019) and low self-esteem (P<0.001). Multivariate analysis using binary logistic regression revealed that medium to high socio-economic level, unsatisfactory parent-child communication, absence of leisure activities and significant symptoms of social phobia were independent predictors of Internet addiction in children with depression.

**Conclusions:**

Our study highlighted the high frequency of IA in children with depression and demonstrated the implication of certain variables such as medium to high socio-economic status, disruption of the family environment, anxiety and low self-esteem. The identification of these different factors would make it possible to identify a group at risk of IA. This raises the case for introducing prevention and awareness-raising campaigns on IA among depressed children, targeting these groups and targeting health professionals and parents.

**Disclosure of Interest:**

None Declared

